# Development and clinical application of a rapid SARS‐CoV‐2 antibody test strip: A multi‐center assessment across China

**DOI:** 10.1002/jcla.23619

**Published:** 2020-10-16

**Authors:** Mengting Liao, Jiawei Yan, Xinfeng Wang, Huimin Qian, Chao Wang, Dan Xu, Bin Wang, Bo Yang, Shaohui Liu, Mao Zhou, Qian Gao, Qian Zhou, Jiquan Luo, Zongxiang Li, Wenen Liu

**Affiliations:** ^1^ Health Management Center Xiangya Hospital of Central South University Changsha China; ^2^ Infectious Diseases Hospital of Xuzhou City Xuzhou China; ^3^ Chest Hospital of Shandong Province Jinan China; ^4^ The Center for Disease Control and Prevention of Jiangsu Province Nanjing China; ^5^ Eye Center of Xiangya Hospital Central South University Changsha China; ^6^ Department of Clinical Laboratory the First Hospital of Changsha Changsha China; ^7^ Department of Clinical Laboratory Xiangya Hospital of Central South University Changsha China; ^8^ Sinocare Inc. Changsha China

**Keywords:** colloidal gold immunochromatography assay, COVID‐19, multi‐center assessment, performance evaluation, SARS‐CoV‐2, serological test

## Abstract

**Background:**

The ongoing coronavirus disease 19 (COVID‐19) is posing a threat to the public health globally. Serological test for SARS‐CoV‐2 antibody can improve early diagnosis of COVID‐19 and serves as a valuable supplement to RNA detection.

**Method:**

A SARS‐CoV‐2 IgG/IgM combined antibody test strip based on colloidal gold immunochromatography assay was developed, with both spike protein and nucleocapsid protein of SARS‐CoV‐2 antigen used for antibody detection. From 3 medical institutions across China, serum or plasma of 170 patients with confirmed COVID‐19 diagnosis and 300 normal controls were collected and tested with the strip. Sensitivity, specificity, kappa coefficient, receiver operating characteristic (ROC) curve, and area under the curve (AUC) were analyzed. Positive rates in different medical centers, age group, gender, and different disease course were compared.

**Results:**

158 out 170 samples from confirmed COVID‐19 patients had positive results from the test, and 296 out of 300 samples from normal controls had negative results. The kit was 92.9% sensitive and 98.7% specific. The positive rate was 77.3% during the first week after disease onset, but reached 100% since day 9. AUC and kappa coefficient were 0.958 and 0.926, respectively, which showed the consistency of the test results with the standard diagnosis. Age or gender caused little variations in the kit sensitivity.

**Conclusion:**

The rapid, easy‐to‐use SARS‐CoV‐2 IgG/IgM combined antibody test kit has a superior performance, which can help with accurate diagnosis and thus timely treatment and isolation of COVID‐19 patients, that contributes to the better control of the global pandemic.

## Graphical abstract

A SARS‐CoV‐2 IgG/IgM combined antibody test strip based on immunochromatography assay was developed with both spike protein and nucleocapsid protein used for detection. With clinical assessment from 3 medical centers from across China, the kit was 92.9% sensitive and 98.7% specific. The positive rate was 77.3% during the first week after disease onset and reached 100% since day 9.

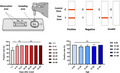

## 1 INTRODUCTION

Coronavirus disease 19 (COVID‐19) caused by severe acute respiratory syndrome coronavirus 2 (SARS‐CoV‐2) is rapidly spreading on a global scale and poses a grave threat to the public health. As a highly contagious disease, it caused over 26,000,000 confirmed cases of infection and over 870,000 deaths as of early September 2020, which are still on a constant rise.^1^ Though the mortality of COVID‐19 is around only 3.3%, the various ways of transmission including droplets, aerosol and fecal transmissions, as well as the large population base of patients, make it urgent to develop approaches to early identifying and controlling the disease.^2^, ^3^, ^4^

The diagnosis of COVID‐19 has been largely dependent on nucleic acid RT‐PCR with patient throat swab. However, the sensitivity of this test is insufficient, due to the low viral loads in upper respiratory tract of patients, failure to correctly collect high‐quality swab specimen, and varied viral loads in different stage of infection. Liang C, et al reported that out of 22 suspected patients, 8 patients had positive results only after 2 consecutively negative results, and 3 patients turned positive after 3 consecutively negative tests.^5^ Delayed diagnosis of COVID‐19 would challenge the timely life‐support treatment, and more seriously, the patient isolation. Alternatively, it was proposed to test the specific antibody in the patient blood as a supplement to nucleic acid RT‐PCR.^6^ IgM antibody can be detected 3 days after the illness onset and lasts until the patient recovers; IgG antibody appears on day 3‐7 and exists constantly even in the convalescence.^7^, ^8^, ^9^ Another report which compared different approaches of detections showed that the total sensitivity of RT‐PCR was 67.1%, while the antibody tests had a sensitivity of 93.1%. Surprisingly, it was up to 99.4% sensitive when combining antibody test with nucleic acid RT‐PCR results.^6^ With the emphasis on the antibody test, the WHO interim guidelines updated on 19th of March approved the need for antibody testing to support COVID‐19 diagnosis.^10^ Accordingly, the 7 edition of guidelines for diagnosis and treatment of COVID‐19 published by National Health Committee of China has included serological test of specific antibody of SARS‐CoV‐2 in the diagnosis criteria, which suggests that in case where a suspected patient gets negative result from PCR, a positive serological antibody test serves as an evidence to confirm the diagnosis.^11^ Further, COVID‐19 vaccine is under rapid development and regarded as a priority for ending the pandemic, which again indicates the importance of antibody detection, as it is needed for the evaluation of response to the vaccine candidates.^12^, ^13^

There has been quite a few detection kits for serological test of SARS‐CoV‐2 antibody.^14^, ^15^, ^16^, ^17^ However, using a single protein as the probe limited the detection sensitivity. Spike (S) protein is located on the surface and able to bind with host cell membranes. Nucleocapsid (N) protein is an abundant protein which structurally binds to RNA.^18^, ^19^, ^20^ Both S and N proteins are highly immunogenic and antibodies against these two can be detected in patients with COVID‐19, but most serological test kits target on either S or N protein alone.^15^ In addition to this, IgG or IgM detection does not work as good as combined IgG/IgM detection. Here we developed a SARS‐CoV‐2 antibody test strip based on colloidal gold and immunochromatography assay, with using both spike protein and nucleocapsid protein of SARS‐CoV‐2 as antigen, which detects both IgG and IgM antibody instead of only one of them. A multi‐center assessment across China was carried out on 470 individuals, consisting of 170 confirmed COVID‐19 patients (by RT‐PCR and clinical manifestations) and 300 negative controls. With this rapid and sensitive SARS‐CoV‐2 IgG/IgM combined antibody test kit, we hope to further improve the accuracy of diagnosis for COVID‐19 patients, thus contributing to better control of the worldwide pandemic.

## 2 MATERIALS AND METHODS

### 2.1 Materials for the test strip

The recombinant antigens of SARS‐CoV‐2 were commercially purchased—S protein (S1‐RBD) from GenScript Inc (Cat#: T80302) and N protein from Fapon Biotech Inc (Cat#: ncov‐PS‐Ag6). Both S protein and N protein were used as the antigen in the kit. Detail information including protein sequence is available on NCBI website: https://www.ncbi.nlm.nih.gov/protein/YP_009724390 for S protein, and https://www.ncbi.nlm.nih.gov/protein/1798174255 for N protein. Mouse anti‐human IgG monoclonal antibody was purchased from GenScript Inc (Cat#: V90401); mouse anti‐human IgM monoclonal antibody was purchased from Clongene Biotech Co. (Cat#: MS00704). Chicken IgY (Kitgen Bio‐tech Co., Cat#: A108) and goat anti‐chicken IgY polyclonal antibody (Kitgen Bio‐tech Co., Cat#: B108) were used in the control setting. Other materials including fiberglass sample pad (Shanghai Kinbio Tech. Co), nitrocellulose membrane (Sartorius, Cat#: 1UN14E), conjugate pad (Ahlstrom), PVC panel (Shanghai JN Bio) were all purchased from commercial companies.

### 2.2 Preparation of colloidal gold and conjugation of SARS‐CoV‐2 antigen

Purified water was brought to boil in a round‐bottom flask, when reductive agent trisodium citrate dihydrate solution was added and kept boiling for 1 minute. Tetrachloroauric acid solution was then added and kept boiling for 10 minutes. The solution was cooled down to room temperature, and purified water was added to make 800 milliliter of total volume. The final colloidal gold solution was checked, and those with good permeability under the light were deemed qualified. Collect the solution in a clean flask until use.

Potassium carbonate was added to colloidal gold solution and stirred for 20 minutes. While stirring, N protein of SARS‐CoV‐2 antigen was added to make final concentration of 10μg/mL and kept stirring for 45 minutes. Centrifuge at 12 000 rpm for 50 minutes and remove the supernatant. Collect the conjugation solution, centrifuge again, and remove the supernatant, and the final colloidal gold conjugated with SARS‐CoV‐2 N protein was obtained. Apply the same protocol to make colloidal gold conjugated with SARS‐CoV‐2 S protein and with goat anti‐chicken IgY polyclonal antibody. Mix all the conjugates and spray to the conjugate pad.

### 2.3 Test principal of SARS‐CoV‐2 antibody test strip

The SARS‐CoV‐2 antibody test strip was based on colloidal gold immunochromatography assay. Colloidal gold conjugated with recombinant SARS‐CoV‐2 antigen and goat anti‐chicken IgY was coated in reagent conjugate pad. Mouse anti‐human IgG monoclonal antibody and mouse anti‐human IgM monoclonal antibody were coated in the detection area of nitrocellulose membrane. Chicken IgY was coated in the control area. During the test, specific SARS‐CoV‐2 antibody in the sample combines to the colloidal gold conjugated with recombinant SARS‐CoV‐2 antigen and forms an immune complex, which flows across the membrane due to capillary action. If the sample contains specific SARS‐CoV‐2 antibody (IgG or IgM), it will be captured by the mouse anti‐human monoclonal antibody in the test area and form a visible line (test line). Excess colloidal gold conjugated with goat anti‐chicken IgY polyclonal antibody keeps moving and specifically binds with the chicken IgY in the control area and from a visible line (control line). If the sample does not contain SARS‐CoV‐2 antibody, only control line will appear.

### 2.4 Collection of patient samples

Serum or plasma samples from 470 participants including 170 confirmed COVID‐19 patients and 300 excluded cases were collected from 3 centers across China, which were 1) The Center for Disease Control and Prevention of Jiangsu Province, 2) Chest Hospital of Shandong Province, and 3) Infectious Diseases Hospital of Xuzhou City. The sample collection was performed by following the “SARS‐CoV‐2 infected pneumonia laboratory test technical guidance” protection requirement. Confirmed cases were defined based on the diagnostic criteria including nucleic acid RT‐PCR and clinical characteristics, according to the Guideline of Diagnosis and Treatment of COVID‐19 published by National Health Committee of China.^21^ Excluded cases included 1) close contacts of confirmed COVID‐19 patients who were excluded for COVID‐19 diagnosis, 2) fever patients with other diseases, 3) patients with other pulmonary diseases. Clinical information including the duration between disease onset to serological test and clinical diagnosis were all collected. This study was approved by the ethics committee of The Center for Disease Control and Prevention of Jiangsu Province, Chest Hospital of Shandong Province, and Infectious Diseases Hospital of Xuzhou City. Serum and plasma samples were stored at 2 ‐ 8℃ within 7 days or −70℃ within 6 months before testing. Samples have not gone through heat inactivation as it interferes with the immunoreaction of antibodies to SARS‐CoV‐2.^22^

### 2.5 Sample testing

A dropper was inserted the into the blood collection tube and absorbed the sample automatically until it reached to 10μL. The sample was added to the sampling area on the strip, and 2 drops of sample diluent were added (Figure S1A). 15‐20 minutes later, the results were read. The interpretation of the results is shown in Figure S1B.

### 2.6 Data analysis

From patients with detailed clinical information, a timeline graph was illustrated by Office PowerPoint to show disease process of each patient, as well as the timepoint and result of antibody test.

For all participants from three medical centers, detection kit sensitivity, specificity, kappa evaluation, receiver operation characteristic curve (ROC curve), and area under the curve (AUC) were generated with IBM SPSS Software (Ver. 23) to assess the accordance of the test result and the clinical diagnosis.

Sensitivity, specificity, Youden index, positive predictive value, and negative predictive value were calculated according to formula below:

Sensitivity = true positive/ (true positive + false negative).

Specificity = true negative/ (true negative + false positive).

Youden index = sensitivity +specificity −1.

Positive predictive value = true positive/ (true positive + false positive).

Negative predictive value = true negative/ (true negative + false negative).

With clinical information for each patient, dynamic analysis of positive rate was performed. Positive rates for different clinical centers, gender, age group, and different disease course were further depicted and analyzed by GraphPad Prism 8.0 with unpaired t test, where *P* < .05 was considered statistically significant.

## 3 RESULTS

### 3.1 Production of SARS‐CoV‐2 antibody test strip and result display of clinical samples

**Figure 1:**
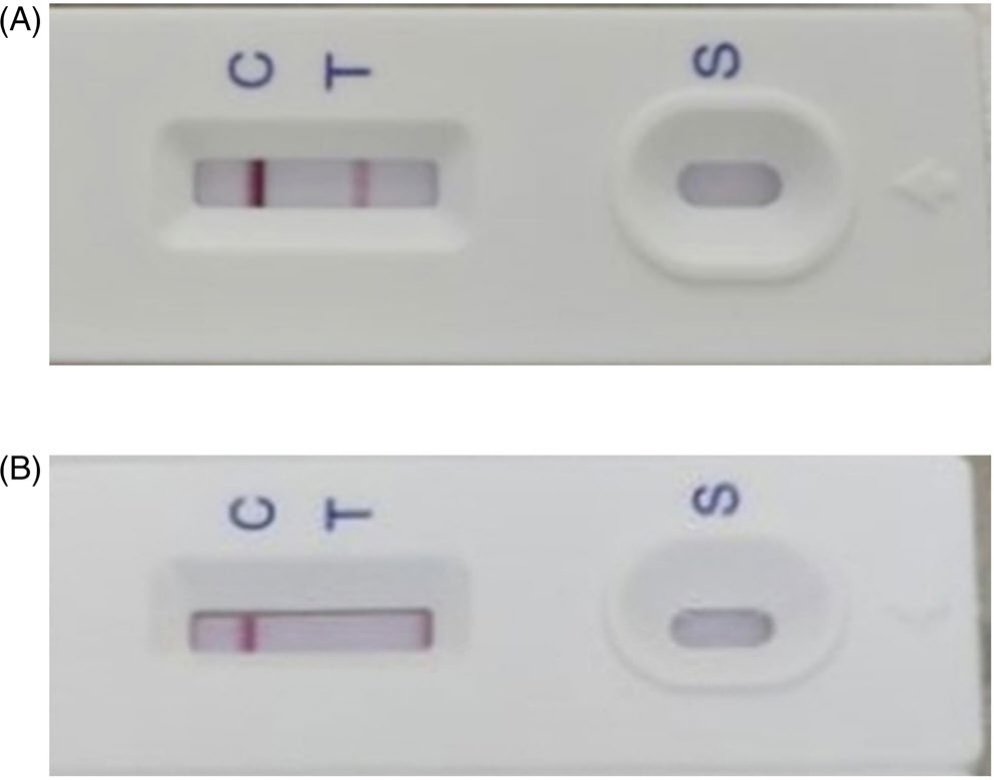
Examples of results from clinical samples. (A) An example of positive result. (B) An example of negative result. S: sampling area; T: test line; C: control line

The product of test strip for specific SARS‐CoV‐2 antibody based on colloidal gold and immunochromatography assay was completed by Changsha Sinocare Inc, China. To test the strip, serum or plasma from 170 confirmed COVID‐19 patients and 300 normal controls were collected. The appearance of both control line and test line on the strip was considered as a positive result, suggesting that IgM or IgG or both were present in the sample. The appearance of only control line was considered as negative, indicating that none of IgM or IgG was present in the sample. An example of both positive and negative result is shown in Figure 1A,B.

### 3.2 Demographic information of participants for clinical assessment

The clinical information of these participants is summarized in Table 1. The 170 COVID‐19 patients consisted of 87 males and 83 females, with an average age of 45.4, 44 of them received the serological test by out kit within 7 days after illness onset, 30 received the test after 8‐14 days after onset, 23, 21, 20, 21, 9, and 2 patients were tested after 15‐21, 22‐28, 29‐35, 36‐42, 43‐49, 50‐56 days after illness onset, respectively. As for clinical classification of these 170 patients, there were 9 patients with mild, 41 with moderate, 3 with severe, and 7 with critical pneumonia, and 108 patients were unknown due to limited information availability. The 300 individuals for normal controls included 194 males and 106 females with an average age of 41.4. Among them, there were 126 patients who had pulmonary infection by diseases other than COVID‐19, 49 patients with fever of unknown origin at admission, 98 participants who had close contact with COVID‐19 patients, and 27 individuals with other reasons to have serological test.

**Table 1:** Summary of clinical characteristics of patients with COVID‐19 and control

	COVID‐19 confirmed patients n = 170	Normal controls n = 300
Age, years (mean ± SD)	45.4 ± 17.1	41.4 ± 19.2
Gender (n, %)		
Male	87 (51.2%)	194 (64.7%)
Female	83 (48.8%)	106 (35.3%)
Sample Type (n, %)		
Serum	166 (97.6%)	258 (86.0%)
Plasma	4 (2.4%)	42 (14.0%)
Diagnosis or reason to be tested (n, %)		
COVID‐19	170 (100%)	0 (0%)
Pulmonary infection by other causes	0 (0%)	126 (42.0%)
Fever of unknown origin	0 (0%)	49 (16.3%)
Close contacts of COVID‐19 patients	0 (0%)	98 (32.7%)
Others	0 (0%)	27 (9.0%)
Days after illness onset, n (%)		
1‐7 days	44 (25.8%)	N.A
8‐14 days	30 (17.6%)	N.A
15‐21 days	23 (13.5%)	N.A
22‐28 days	21 (12.4%)	N.A
29‐35 days	20 (11.8%)	N.A
36‐42 days	21 (12.4%)	N.A
43‐49 days	9 (5.3%)	N.A
50‐56 days	2 (1.2%)	N.A
Clinical classification		
Mild	9 (5.3%)	N.A
Moderate	41 (24.1%)	N.A
Severe	3 (1.8%)	N.A
Critical	7 (4.1%)	N.A
Unknown	108 (63.5%)	N.A

### 3.3 Evaluation of SARS‐CoV‐2 antibody kit performance

**Figure 2:**
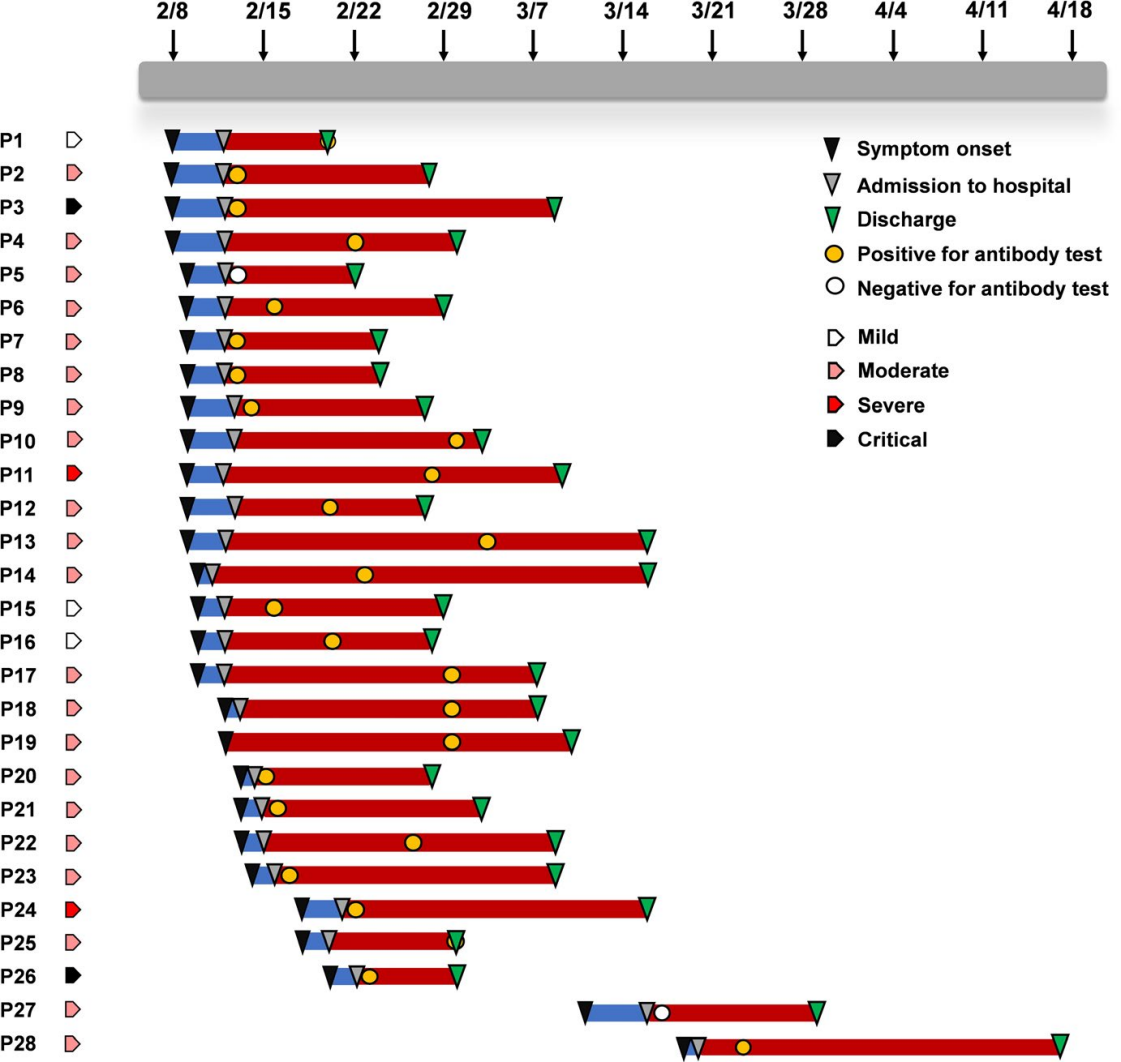
Timeline graph of 28 COVID‐19 patients. A timeline was depicted to show the process of symptom onset, admission, and discharge of 28 patients; the timepoint of antibody test by our kit was also marked. Most patients were positive for the test except for 2 patients who received the test within a week after symptom onset

Of the patients with detailed information, a timeline graph was depicted to illustrate the illness onset, hospital admission, serological test for antibody, and discharge of each patient, along with their clinical classification (Figure 2). In all the 28 patients, most were positive for antibody test except for 2 patients who received the test within a week after symptom onset.

Among the total 170 samples with confirmed cases from all medical centers, 158 had positive result from the test, generating a sensitivity of 92.9%. Among the 300 samples from normal controls, 296 had a negative result, with a specificity of 98.7% (Table 2). From the Center for Disease Control and Prevention of Jiangsu Province, Chest Hospital of Shandong Province and Infectious Diseases Hospital of Xuzhou City, the sensitivity were 97.6%, 87.3%, and 95.4%, respectively, and the specificity was 96.9%, 100%, and 99.1%, respectively. Further, to evaluate the consistency of the test result and the standard diagnosis, Youden index, positive predictive value, negative predictive value, and kappa coefficient were calculated for all samples and each center (Table 2). ROC curve for all participants was obtained and shown in Figure S2A, and ROC curves for each medical center were shown in Figure S2B‐D. AUC and kappa coefficient were summarized as well (Table 2). These data verified that the SARS‐CoV‐2 antibody test strip that we developed had a considerable positive rate, and the result was highly consistent with the diagnosis by the standard criteria.

**Table 2:** Results of SARS‐CoV‐2 antibody test strip in all centers

Center	Summary of all centers		CDCJS		CHSD		IDHXZ	
Group	Confirmed cases	Normal controls	Confirmed cases	Normal controls	Confirmed cases	Normal controls	Confirmed cases	Normal controls
Total (n)	170	300	42	98	63	89	65	113
Positive (n, %)	158 (92.9%)	4 (1.3%)	41 (97.6%)	3 (3.1%)	55 (87.3%)	0 (0%)	62 (95.4%)	1 (0.9%)
Negative (n, %)	12 (7.1%)	296 (98.7%)	1 (2.4%)	95 (96.9%)	8 (12.7%)	89 (100%)	3 (4.6%)	112 (99.1%)
Sensitivity (95%CI)	92.9% (89.1%‐96.8%)		97.6% (92.8%‐100.0%)		87.5% (79.2%‐95.8%)		95.3% (90.0%‐100.0%)	
Specificity (95%CI)	98.7% (97.4%‐100.0%)		96.9% (93.5%‐100.0%)		100.0% (100.0%‐100.0%)		99.1% (97.4%‐100.0%)	
Youden index (%)	91.6		94.5		87.3		94.5	
Positive predictive value (%)	97.5		93.2		100.0		98.4	
Negative predictive value (%)	96.1		99.0		91.8		97.4	
AUC (95%CI)	0.958 (0.934‐0.982)		0.973 (0.940‐1.000)		0.935 (0.886‐0.985)		0.972 (0.941‐1.000)	
Kappa	0.926		0.933		0.890		0.951	

Note

Abbreviations: CDCJS, The Center for Disease Control and Prevention of Jiangsu Province; CHSD, Chest Hospital of Shandong Province; IDHXZ, Infectious Diseases Hospital of Xuzhou City.

**Figure 3:**
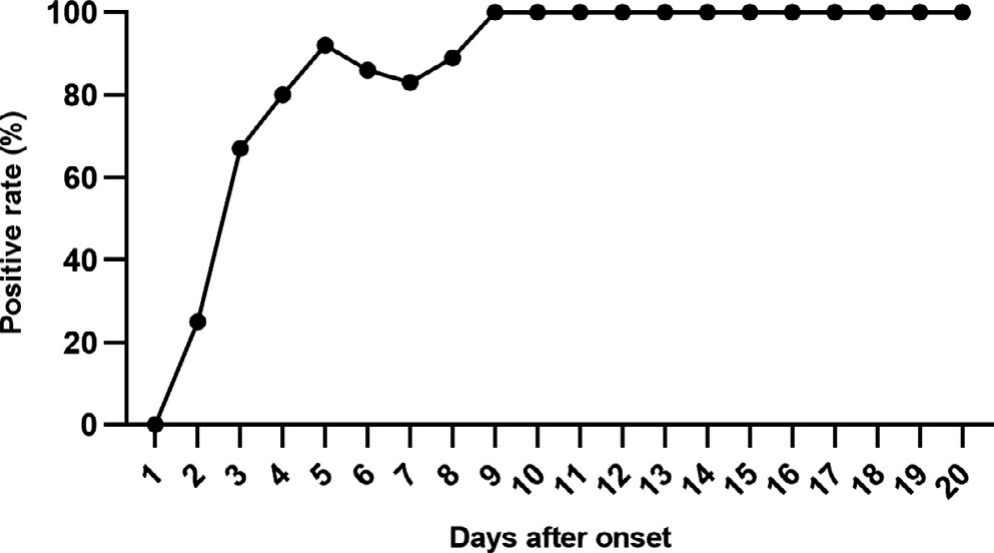
Dynamic changes of positive rates of the test kit. The positive rate increased within the first week after illness onset, and reached 100% on day 9

**Figure 4:**
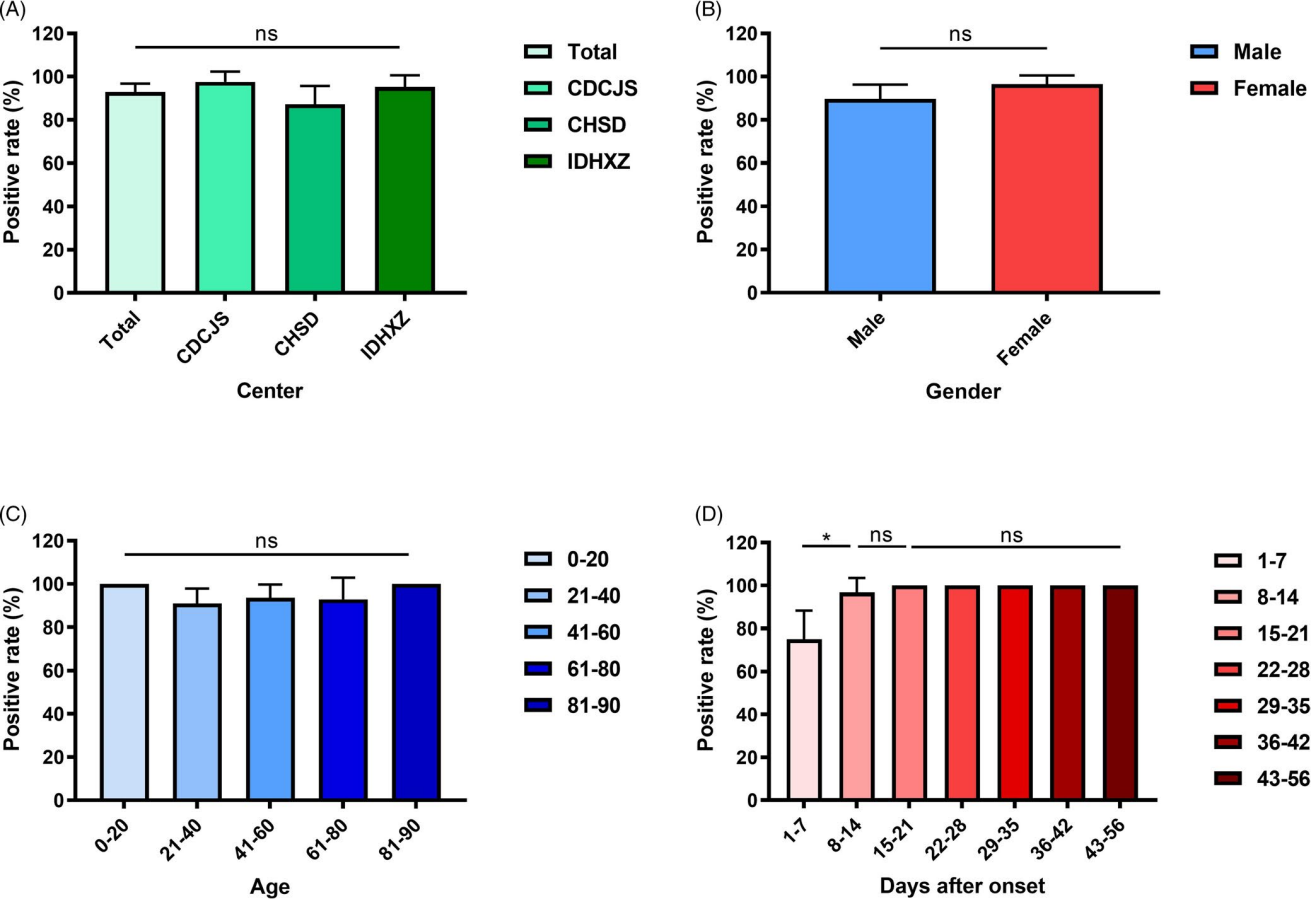
Sensitivity comparison among results from different patient groups. Column chart that compares sensitivity in different medical centers (A), gender (B), age group (C) and disease course (D). Data were shown as mean with 95% confidential interval (CI). *P* < .05 was considered as statistically significant. CDCJS: The Center for Disease Control and Prevention of Jiangsu Province; CHSD: Chest Hospital of Shandong Province; IDHXZ: Infectious Diseases Hospital of Xuzhou City

Despite analysis in total participants, the dynamic changes of positive rates were depicted with the information on disease course of each patient. The positive rate increased during the first week after illness onset, reached 100% on day 9 and remained unchanged (Figure 3). Further, the kit sensitivity in different medical centers, gender, age, and disease course was compared, and little variation with kit sensitivity was found (Figure 4A‐C), except for that it was lower for samples collected within 1‐7 days after symptom onset (Figure 4D).

## 4 DISCUSSION

In this study, we developed a rapid, convenient, and easy‐to‐interpret kit for SARS‐CoV‐2 specific IgG/IgM antibody. With these advantages, the test kit can be applied for early diagnosis of SARS‐CoV‐2 infection among the suspected patients, medical staff, and population with possible exposure history, which ensures they get the treatment and isolation in time. Despite some argument that the antibody kit had limited efficacy in community screening, it is officially recommended by WHO as a diagnostic tool for COVID‐19.^10^, ^23^

Another application of antibody test is the treatment‐related evaluations. COVID‐19 vaccine is the most attractive approach to eradicate the virus globally, and it is moving toward the preclinical and clinical trials.^24^, ^25^ As active immunization generated, the serological antibody can be detected, which indicates the immunity against SARS‐CoV‐2 infection. Additionally, monoclonal antibody therapy was raised as a promising intervention for COVID‐19, and the transfusion of convalescent plasma was proved to be beneficial in critically ill patients.^26^, ^27^, ^28^, ^29^ This also suggested the need for antibody test as the convalescent plasma should be assessed before the transfusion was carried out.

Further, the serological test is potentially useful to identify asymptomatic infection. There is a largely unknown but presumably high proportion of asymptomatic carriers of SARS‐CoV‐2. A recent report showed 12% of asymptomatic (SARS‐CoV‐2 detected but symptoms never develop) and 30% of presymptomatic cases (SARS‐CoV‐2 detected before symptom onset) in travelers and returning residents to Brunei.^30^ These populations can carry the virus around and contribute to the spread among more people, according to epidemiologic, virologic, and modeling studies.^31^, ^32^, ^33^ It was proposed that an effective way to cutoff the transmission is to scale up the serological test for antibody.^33^ However, it remains controversial whether asymptomatic cases generate the immune response that is detectable by serological test for antibody.

There has been other report on clinical evaluation of serological test kit for SARS‐CoV‐2. Lijia et al assessed a quantitative test kit for IgG and IgM, but only with a small group of patients (15 COVID‐19 patients and 50 normal controls).^34^ Li et al developed a combined IgG/IgM antibody kit just as we did in the present study, but their overall sensitivity was only 88.66% and specificity was 90.63%, which were both lower than results from our study.^17^ We tested our kit with samples collected from three different medical institutions, which makes the results more convincing and reliable. Our kit reached a total sensitivity of 92.9% and specificity of 98.7%, with little variation between different centers, age group, and gender, indicating that this kit has considerable value in clinical practice.

Admittedly, there are some limitations of the antibody test kit. First, our IgG/IgM combined kit does not distinguish IgG and IgM apart, which failed to inform the stage of infection, but on the other hand, it tends to avoid false‐negative result and have higher sensitivity. Second, same as other kits, the result cannot be used alone for decision making in the treatment or other management, but should be combined with nucleic acid test, clinical presentations, or epidemiological contact history.

In conclusion, we developed a rapid and easy‐to‐use SARS‐CoV‐2 antibody test kit which is highly sensitive and specific in the diagnosis for COVID‐19. With the widespread use of the kit, earlier treatment and timely isolation can be achieved, that improves the outcome and controls the extensive transmission of the global pandemic.

## CONFLICTS OF INTEREST

The authors declare no conflicts of interest.

## AUTHOR CONTRIBUTION

WL designed the study. ML, JY, XW, and HQ collected and processed the patient samples and tested the samples. JL and ZL performed the optimization of test kit. ML, CW, BY, BW and SL analyzed and interpreted the data. ML and CW drafted the manuscript. SL, WL, DX, QZ, MZ, and QG revised the manuscript. All the authors approved the final manuscript.

## Supporting information

Figure S1

Figure S2
