# Supplementary figures and images for: Development and clinical application of a rapid SARS‐CoV‐2 antibody test strip: A multi‐center assessment across China
Source: J Clin Lab Anal. 2020 Oct 16;35(1):e23619. doi: 10.1002/jcla.23619 (PMC7645889; doi:10.1002/jcla.23619)

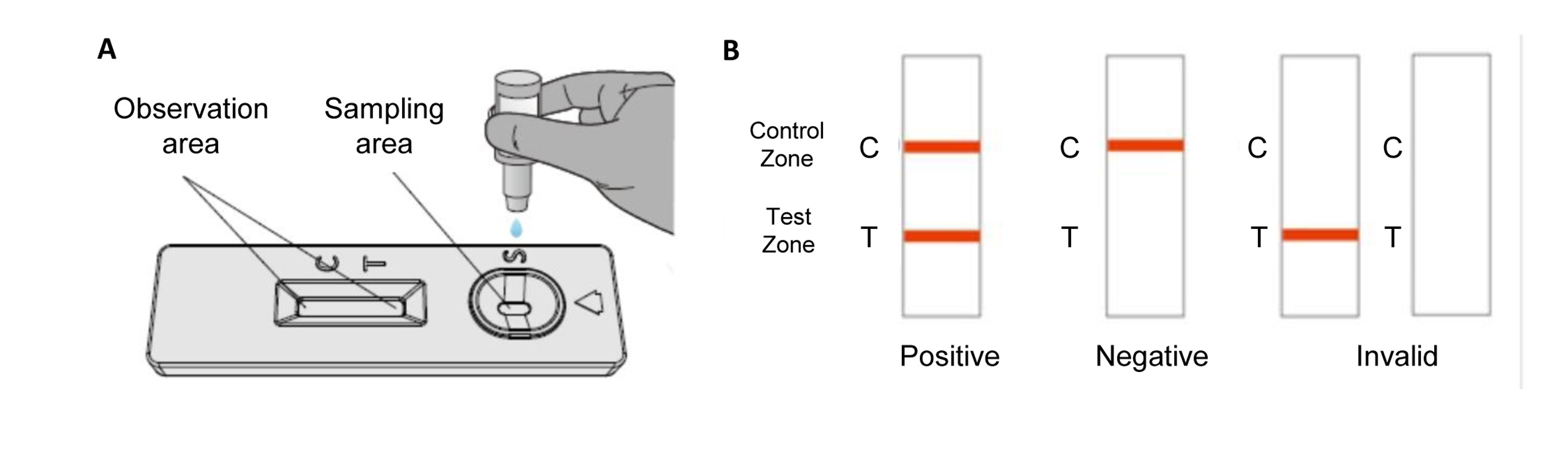

Supplement: Supplementary file 1 — Figure S1 [file JCLA-35-e23619-s001.jpg]

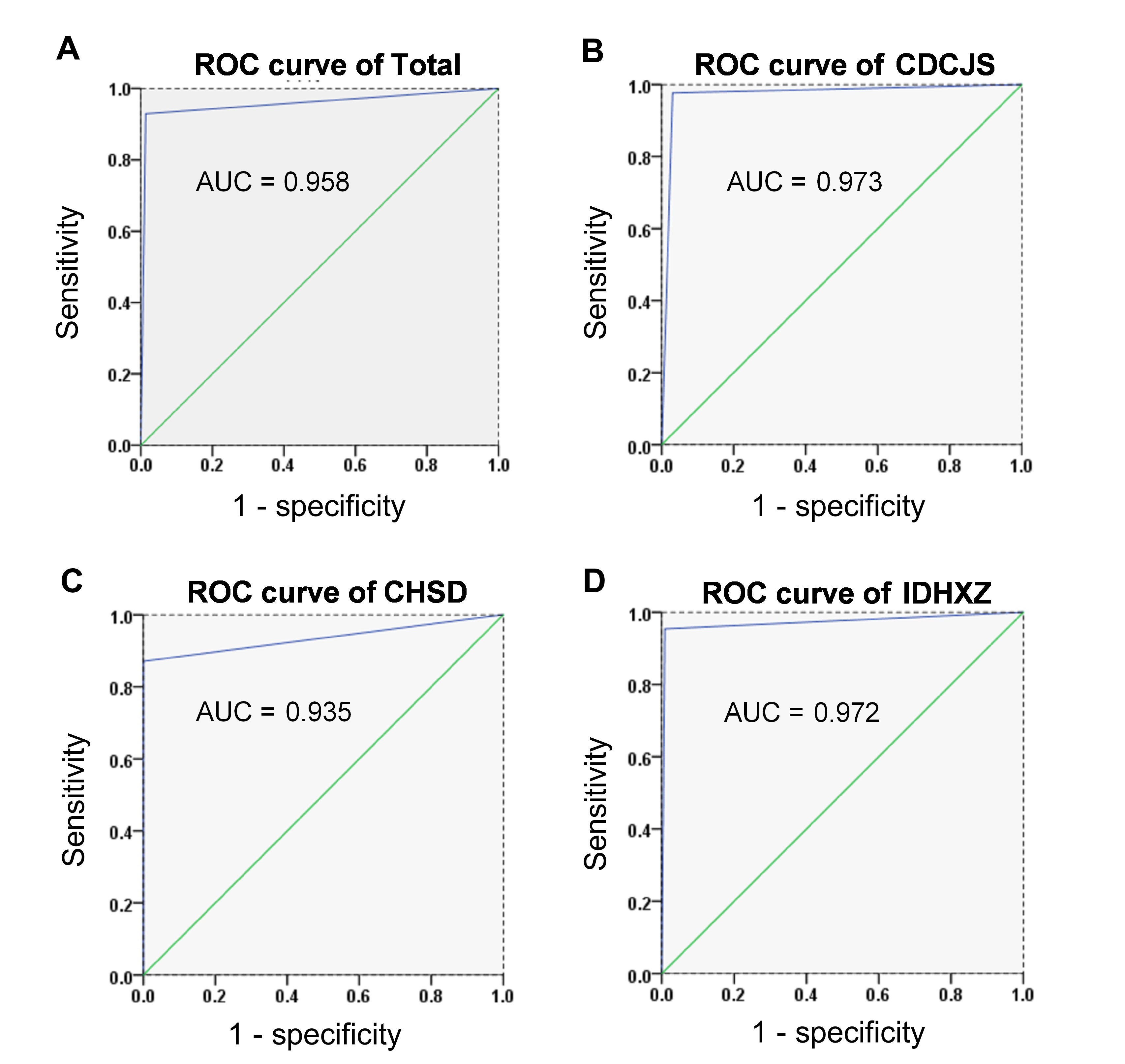

Supplement: Supplementary file 2 — Figure S2 [file JCLA-35-e23619-s002.jpg]
